# Daily Rhythms in Mosquitoes and Their Consequences for Malaria Transmission

**DOI:** 10.3390/insects7020014

**Published:** 2016-04-14

**Authors:** Samuel S. C. Rund, Aidan J. O’Donnell, James E. Gentile, Sarah E. Reece

**Affiliations:** 1 Centre for Immunity, Infection and Evolution, University of Edinburgh, Edinburgh EH9 3FL, UK; 2 Institutes of Evolutionary Biology, and Immunology and Infection Research, University of Edinburgh, Edinburgh EH9 3FL, UK; Aidan.Odonnell@ed.ac.uk (A.J.O.D.); Sarah.Reece@ed.ac.uk (S.E.R.); 3 Invincea Labs LLC, Arlington, VA 22203, USA; jegentile@gmail.com

**Keywords:** *Anopheles*, chronobiology, circadian, diel, diurnal, nocturnal, *Plasmodium*

## Abstract

The 24-h day involves cycles in environmental factors that impact organismal fitness. This is thought to select for organisms to regulate their temporal biology accordingly, through circadian and diel rhythms. In addition to rhythms in abiotic factors (such as light and temperature), biotic factors, including ecological interactions, also follow daily cycles. How daily rhythms shape, and are shaped by, interactions between organisms is poorly understood. Here, we review an emerging area, namely the causes and consequences of daily rhythms in the interactions between vectors, their hosts and the parasites they transmit. We focus on mosquitoes, malaria parasites and vertebrate hosts, because this system offers the opportunity to integrate from genetic and molecular mechanisms to population dynamics and because disrupting rhythms offers a novel avenue for disease control.

## 1. Introduction

The 24-h day involves cycles of predictable environmental changes that include rhythms in ambient light, temperature, humidity, UV radiation and resource availability. Organisms are assumed to have evolved mechanisms to respond directly to both the daily light:dark cycle, as well as molecular circadian clock mechanisms to anticipate daily rhythms in environmental factors that influence fitness and organize their physiology and behaviors in accordance [1]. These rhythms are colloquially and collectively known as “circadian rhythms.” However, they include: (1) daily rhythms observed only under light:dark conditions (“diel rhythms”), which may or may not be endogenously driven by a molecular circadian clock; and (2) endogenously-regulated rhythms observed under constant environmental conditions (true “circadian rhythms”). Consequently, a great diversity of biological processes are rhythmically regulated, and fitness is reduced if there is a divergence between the environmental rhythm and the rhythm of the organism [2,3,4]. An example of adaptive time-of-day regulated biology in some insects includes minimizing the risk of desiccation by being nocturnal because environmental humidity is higher at night than in the day [5]. Daily rhythms also occur in interactions between organisms and are likely to be shaped by coevolution. This includes predation risk, reproductive opportunities and, for parasites, opportunities to transmit to new hosts. For example, if pathogens are acquired during foraging, then it may be advantageous to forage at times-of-day when pathogens are least infective. This, in turn, may result in selection on the pathogen to coincide maximum infectivity with host foraging activity [6,7,8,9,10,11,12].

The molecular workings of clock mechanisms in model systems are well understood. The classical circadian clock is cell autonomous and at the molecular level is comprised of a series of transcriptional-translational feedback loops (TTFLs) that have three key attributes: (1) completion takes approximately 24 h; (2) they persist in the absence of any entraining cues, such as light; and (3) they are resistant to changes in temperature [1]. TTFL-based molecular clocks have been described across diverse taxonomic groups (including cyanobacteria, plants, yeast, mammals and insects [13]), but share little homology in the genes that underpin them. Recently, the existence of a non-transcriptional, widely-conserved and more ancient clock mechanism was reported [14,15].

Work in many groups has revealed that the molecular clocks of *Anopheles gambiae*, *Aedes aegypti* and *Culex quinquefasciatus* mosquitoes more closely resemble the clock of the butterfly than *Drosophila melanogaster*. The canonical clock genes, *clock*, *period*, *timeless*, *cryptochrome 2*, *PAR-domain protein 1* and *cycle*, are rhythmically expressed. *Cryptochrome 2*, *timeless* and *period* share similar nocturnal peak phases and are anti-phasic with the day-time peaks in the expression of *clock* and *cycle* [16,17,18,19,20]. From work in *An. gambiae*, it appears that mosquito Cryptochrome 1 functions as a circadian photoreceptor (“*Drosophila*-like”), but unlike *Drosophila*, it is not rhythmically expressed, whereas Cryptochrome 2 appears to function as a transcriptional repressor in the molecular clock mechanism (“mammalian-like”) [16,17,18].

Given that circadian clocks play a key role in non-infectious human diseases (e.g., jet lag and obesity associated with shift working [4]) and increasing evidence of rhythms in parasite behaviors and host/vector immune responses, we contend that circadian and diel rhythms also shape the severity and transmission of infectious diseases. To illustrate this, we review how rhythms influence the biology of mosquitoes and their interactions with the vertebrate hosts they feed from and the parasites they transmit. We focus on *Anopheline* mosquitoes, human hosts and *Plasmodium* parasites (which cause malaria). *Anophelines* are a widely-studied model system for other mosquito and non-mosquito vectors. We begin by introducing the vectorial capacity equation (which estimates the rate of secondary cases of malaria arising from each infected human host) and discuss the chronobiology of mosquitoes. We then outline the time-of-day-specific biology of interactions between mosquitoes and hosts and parasites and explain how rhythms can be the cause and consequence of competing selection pressures. Finally, we integrate this information into a framework to investigate circadian rhythms in malaria transmission and suggest novel interventions resulting from disrupting rhythms.

## 2. The Chronobiology of Mosquitoes

### 2.1. Anopheles Mosquitoes Live in a Rhythmic World

**Figure 1:**
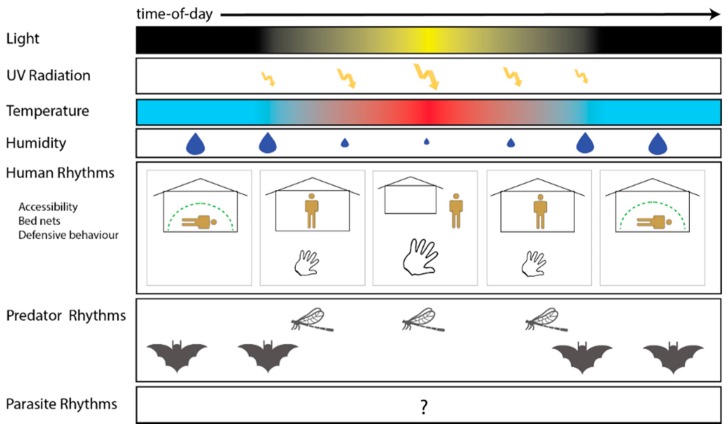
Some of the environmental rhythms a mosquito may be exposed to across the 24-h day. These include physical environmental changes, such as ambient light, UV radiation, temperature and humidity. Mosquitoes also experience the biological rhythms of their hosts, such as when humans are inside or outside of their dwelling, under or not under a bed net and awake or resting (only alert hosts display defensive behavior); and rhythms in predators, such as dragonflies (diurnal) or bats (nocturnal). Parasites may also have rhythms in the activities they undertake in mosquitoes that impact mosquito fitness.

Mosquitoes live in an environment that changes dramatically across the 24-h day (Figure 1) with the most obvious being the environmental light:dark cycle. As a consequence of the rising and the setting of the Sun, there are rhythms in environmental stressors, such as UV radiation and humidity levels (*i.e.*, desiccation risk). The environmental light:dark cycle also drives rhythms that are manifested in how other organisms interact with mosquitoes. For example, there are 24-h rhythms for when human hosts are inside/outside their dwelling, under/not under a bed net and awake/resting (defensive behavior requires an alert host) and for predation risk. Mosquitoes have evolved to temporally regulate a large proportion of their biology, from gene expression, to physiology, to behaviors. The most classical example is the behavioral observation that *Anopheles* spp. malaria mosquitoes are “night biters” and that *Aedes* spp*.* dengue/yellow fever mosquitoes are “day biters” [21,22]. However, the selection pressures resulting in these daily activity patterns are unknown. Mosquitoes temporally regulate more of their biology than simply biting time (see Table 1). This includes rhythms in the expression of thousands of genes, comprising, for example, ~20% of the *An. gambiae* genome and at least ~8% of detectable head transcripts in *Ae. aegypti* [18,20]. Circadian expression profiles of the transcriptomes of these two species can be easily visualized at the Bioclock website developed and maintained by the laboratory of Giles Duffield [23].

**Figure 2:**
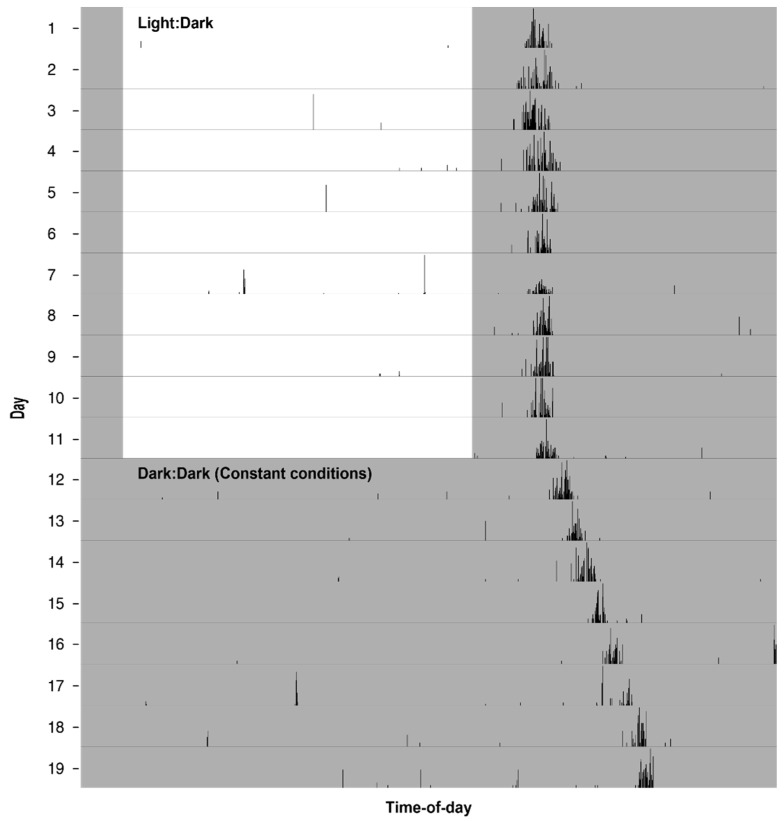
Flight activity rhythm of a single mated *An. stephensi* female mosquito continuously monitored for eleven days in a 12:12 light:dark cycle with 1 h-long dawn and dusk transitions, followed by a further eight days in constant dark conditions. Note, under the entraining conditions of a light:dark cycle, flight activity begins at approximately the same time each day. Under constant dark:dark conditions (where there are no light cues to entrain the clock), flight activity maintains a rhythm driven by its non-entrained, free running circadian clock. In the *An. stephensi* mosquito, the non-entrained clock free runs at a period longer than 24 h as evidenced by flight activity beginning slightly later each day. Recording was performed using a locomotor activity monitor (L.A.M.) from TriKinetics, Inc. (Waltham, MA, USA) at 26 °C and 60% relative humidity. See Rund *et al.* 2012 for the methods [67].

Many of the biological processes highlighted in Table 1 are regulated directly by the circadian clock. Others, however, may be driven in whole or in part by a direct (phenotypically plastic) response to (often rhythmic) environmental stimuli (especially the light:dark cycle). In these instances, if a rhythmic stimulus is taken away, the diel rhythm is dampened or disappears. Aspects of biology that are mainly or entirely under clock control demonstrate a periodic nature even in the absence of environmental rhythms. This contrasts them with simple phenotypic plasticity driving diel rhythms (*i.e*., diel rhythms driven by a cue-response system, not by an endogenous clock). For example, Figure 2 highlights behavioral flight rhythms in *An. stephensi*. Note, under constant conditions (*i.e.*, no environmental rhythms, also known as “free running conditions”), the mosquito still maintains an ~24-h rhythm in flight behavior, anticipating the arrival of night and temporally regulating flight behavior to the expected nighttime. Other mosquito rhythms known to be driven by the circadian clock include gene expression, metabolism, pupation and biting behavior (see Table 1). Divergence from an exactly 24-h rhythm under the constant condition demonstrates that biological clocks may run slightly faster or slower than 24 h. This divergence is not observed in nature, because the clock is normally reset everyday by the light:dark cycle. It is also important to note that organisms do not do all things at all times-of-day. For a mosquito, this likely means they cannot have constitutively high immune responses and reactive oxygen species responses or a constitutive insecticide detoxification response. This is likely due to constraints on resources (conservation of energy) and the fact that some processes are incompatible (e.g., mopping up reactive oxygen species and pathogen defense or opposing biochemical processes) [109].

### 2.2. Behavioral Rhythms

*Anopheles* mosquitoes are primarily night biters ([21,34]; see Table 1). Locomotor flight activity has been monitored in the laboratory in several *Anopheles* species and strains and is similarly nocturnal. These mosquitoes also spontaneously become active at or shortly after dusk ([40,61,62,63,64,66,67]; Figure 2). A pronounced activity peak at the start of the active period occurs in mate-seeking males and females, corresponding with the well-known mating swarm behavior [79]. Following blood feeding, females display similar, but significantly dampened flight rhythms for several days until they oviposit, a behavior timed to the early night [55,56,58,64]. Males also display a daily rhythm in the erection of their antennal hairs, which they use to acoustically locate females in the mating swarm [80]. Differences in the time-of-day that mating swarms are formed by different strains/subspecies have been hypothesized as a prezygotic reproductive barrier contributing to speciation [62,67,79,81] in this very rapidly speciating genus [110]. Other *Anopheles* behaviors, such as pupation and sugar feeding, are also nocturnal activities ([57,65,78,84,88]; see Table 1). Existing in a nocturnal niche minimizes the risks of desiccation and UV damage from the Sun. It also provides protection from some predators; for example, dragonfly predation of *Anopheles freeborni* occurs during daylight hours (dragonflies locate their prey visually) [95]. Rhythmic mosquito behaviors can affect vectorial capacity (*m* and *P* in Box 1). Avoiding predation and desiccation maximizes mosquito longevity, which is a major determinant of opportunities to transmit malaria parasites.

Box 1 **Variants of the vectorial capacity equation for human malaria [111,112] estimate the rate of secondary cases arising from each infected human host.**

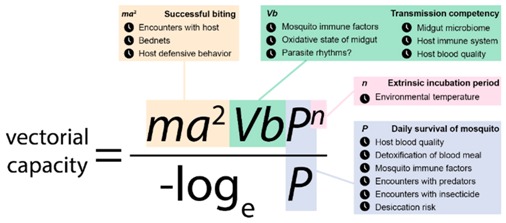

We summarize the parameters involved and illustrate how they are subject to the effects of daily rhythms:

*ma*^2^ (orange): The number of mosquitoes per person (*m*) and their biting rate on humans (*a*). Therefore, there are *ma* bites per host or, considered together, *ma*^2^ can represent the number of mosquitoes that have bit a human twice, once to acquire a malaria infection and once to transmit the parasite. Mosquitoes can only feed on humans during times-of-day when they are foraging *and* hosts are not protected under bed nets [33,35,37,38]. Defensive behavior (“swatting”) by alert hosts in the daytime reduces the success of biting (see Section 2.2, Section 2.3, Section 2.4, Section 2.5, Section 2.6, Section 2.7 and Section 4).

*Vb* (green): Captures the transmission competency for the host and vector. This parameter can be decomposed into the probability of infectious sporozoites successfully developing in the mosquito (*V*) and the probability of a human becoming infected by parasites from that mosquito (*b*). The probability of a mosquito becoming infectious involves many processes that are likely to be rhythmic and impact on the establishment of parasites. For example, there are daily rhythms in the expression of genes involved in immune responses and reactive oxygen species (ROS) detoxification [18] that may result in the mosquito being more susceptible to the parasite at certain times-of-day (see Section 2.7, Section 3.1, Section 3.2, Section 3.3 and Section 4).

*P* (purple): The daily survivability of the mosquito (*P*). Survival rates are influenced by predators, and so, overlap between mosquito and predator foraging times will reduce longevity. Similarly, activity at times-of-day when risk of desiccation is high will decrease longevity (see Section 2.2, Section 2.4, Section 2.5, Section 2.6, Section 2.7, Section 3.1 and Section 4).

*n* (pink): The length of the extrinsic incubation period for parasites to develop into human-infective stages. This is temperature dependent and, thus, sensitive to daily rhythms in environmental temperature [102,103,113] (see Section 3.3).

Humans sleep at night so there is less risk of host defensive behavior (e.g., “swatting”) for nocturnal mosquitoes compared to day-biters. However, at night, humans are more likely to be under protective bed nets than in the daytime. Bed nets can dramatically reduce malaria transmission (especially when insecticide treated), but they do not provide universal protection due to differences in the circadian biology of the mosquito and their human hosts. For example, in the Bolivian Amazon, the malaria vector *Anopheles darlingi* has its peak in biting time several hours prior to the approximate typical human bed time [37]. In northern Ethiopia, 70% of *Anopheles arabiensis* biting activity occurs prior to typical bed time [38]. Finally, Moiroux *et al.*, Geissbühler *et al.* and Matowo *et al.* performed detailed behavioral time-of-day studies of humans and mosquitoes in South Benin (*Anopheles funestus*) and Tanzania (*An. gambiae*, *An. arabiensis*, *An. funestus*, *Culex* spp*.* and *Mansonia* spp*.*)*,* revealing considerable biting risk during times when humans were either outdoors and/or not yet asleep under a bed net [33,35]. The use of bed nets puts selective pressures on mosquitoes to: (1) evolve insecticide resistance; (2) bite at the times-of-day when human hosts are not protected inside a dwelling and/or under bed nets; and/or (3) switch to more accessible host species. Indeed, field reports show that extensive insecticide resistance has occurred [114]; host-preference shifts have been reported upon bed net introduction in many non-African sites [43] (note: African malaria vectors display a very strong host preference towards humans [115,116]). Furthermore, field reports (from as early as 1987) indicate that mosquitoes can shift to day-biting during bed net introduction [36,41,42]. However, a shift in biting times is not always found following bed net introduction [117]. Further work is required to investigate how bed net use interacts with mosquito ecology to shape the evolution of insecticide resistance, foraging rhythms and host preference. For example, recent modeling efforts suggest that varying host densities and sugar-feeding opportunities may shape the development of this biting behavior change [118]. It is also conceivable that human attractiveness to mosquitoes changes in a time-of-day-dependent manner. Mosquitoes locate their hosts by sensing heat, odors and carbon dioxide [119], and body temperature and respiration rate vary during the day in humans [120,121]. Ultimately, the success of mosquitoes at biting humans contributes to the *a* term in the vectorial capacity equation (Box 1).

### 2.3. Physiological Rhythms in Sensory Processes

The transition from day to night involves orders-of-magnitude differences in ambient light, and so, organisms must adjust the sensitivity of their visual systems to cope [122]. In mosquitoes, numerous genes in the visual transduction pathway are rhythmically expressed [18,46] and correspond, for example, to elevated nocturnal rhodopsin levels in the eye [28]. Similarly, because *Anopheles* mosquitoes host-seek at night, they upregulate their olfactory system at night. Gene expression and protein levels of odorant binding proteins (OBPs) peak around dusk, and elevated olfactory sensitivity of hydrophobic compounds to mosquitoes at night has been demonstrated using electroantennograms [18,21]. Producing OBPs involved in host-seeking only at night may be an adaptation to minimize the production of costly proteins or to prevent foraging at dangerous times-of-day (e.g., when the risks of desiccation or host defensive behavior is high). Activity-related mortality contributes to the *m* and *a* terms in the vectorial capacity equation (Box 1). Note, however, that in the case of day-biting mosquitoes, if OBP rhythms have not shifted in tandem, then host detection is impaired, and *a* will be lower.

### 2.4. Rhythms in Insecticide Metabolism

Temporal profiling revealed the expression of rhythmic *An. gambiae* genes encoding enzymes responsible for pyrethroid and/or DDT metabolism (e.g., metabolic resistance). The genes encoding cytochrome P450 mono-oxygenases, CYP6M2, CYP6P3 and CYP6Z1, peaked in expression around dusk, whereas the gene encoding GSTE2 peaked at mid-day [18,24]. Subsequent work revealed that *An. gambiae* CYP6P3 is rhythmic at the protein level [21], and these mosquitoes exhibit a rhythmic metabolic resistance to exposure to the insecticide DDT, with resistance peaking around dusk, and a bimodal rhythm in metabolic resistance to exposure to the insecticide deltamethrin, peaking at early and late day [24]. In *Anopheles arabiensis*, higher resistance to chlorfenapyr challenge during the daytime *versus* nighttime has also been noted [123]. Metabolic resistance is performed by enzymes that breakdown a variety of xenobiotic compounds that insects consume. Thus, it is thought that insects may invest in metabolic detoxification (*i.e.*, have peak resistance to insecticide) at expected feeding times (when xenobiotics would most likely be ingested/digested) [124]. Alternately, it has been proposed that this temporal regulation of metabolic detoxification may instead be important for the degradation of specific byproducts and components of endogenous metabolic processes in a time-of-day-specific manner [24,109]. This hypothesis may explain why not all rhythms in metabolic detoxifying enzymes appear to peak at the same time-of-day. Rhythms in resistance, in conjunction with rhythmic behaviors (e.g., will the mosquito be in a place to encounter the insecticide, at a time the insecticide is present?), will contribute to the ultimate success of insecticide-based interventions. If, as some reports suggest, mosquito behavior is shifting towards day-biting (when bed nets are ineffective), interventions should be utilized that target mosquitoes in these populations regardless of the time-of-day they bite. For example, indoor residual spraying (IRS) targets mosquitoes in their resting places regardless of the time-of-day they rest. Therefore IRS may be a more effective tool than bed nets against day-biting mosquito populations [125,126,127]. Where insecticide fogging is used, timing of application should consider both when the mosquitoes are likely to be in the treatment area and their rhythms in metabolic insecticide resistance [24]. Metabolic insecticide resistance may influence malaria transmission in two ways in the context of vectorial capacity (Box 1): first, by increasing *m* and *a* (if resistance makes bed nets less effective) and, second, by increasing *P* (if resistance enhances survival of treated mosquitoes).

### 2.5. Rhythmic Detoxification of Reactive Oxygen Species

Numerous genes involved in the detoxification of reactive oxygen species (ROS), including *catalase*, are rhythmically expressed in mosquitoes [18]. In *Drosophila*, a similar phenomenon in rhythmic gene expression corresponds to an observed time-of-day-dependent resistance to an ROS challenge [128]. Mosquitoes process their food (blood or plant material) using mechanisms that result in collateral damage (e.g., oxidative stress). The breakdown of blood-hemoglobin, as well as many anti-herbivory defensive molecules releases particularly high levels of ROS [129,130,131]. ROS increases mosquitoes’ post-blood-feed mortality, causes a loss of fecundity (due to physical tissue damage) and alters the midgut microbiome [130,131,132,133]. Because foraging is rhythmic, mosquitoes can anticipate an elevation in ROS levels and use their circadian clock to prepare by upregulating detoxification mechanisms. In contrast to constant upregulation throughout the day, rhythmic upregulation of ROS detoxification mechanisms may minimize the expenditure of resources on costly mechanisms. Furthermore, high levels of ROS favor the melanization of parasites and bacterial clearance [134,135], so ROS detoxification should be minimized at times-of-day when resisting infection is most important. In the context of malaria transmission, parasites and food are acquired simultaneously, so mosquitoes may face a trade-off between ROS detoxification and immune defense. This will affect the *P* and *m* terms in the vectorial capacity equation (Box 1) in opposing ways.

### 2.6. Rhythms in Osmoregulation

Mosquitoes may consume three-times their body weight in blood during a feeding [130], which presents both an enormous osmotic stress and endangers the mosquito due to diminished maneuverability from the added weight [136,137]. Remarkably, the mosquito begins to excrete the excess water before a blood meal is even finished [136]. The vacuolar-type H^+^-ATPase (V-ATPase) plays an important role in mosquito renal function and is enriched in the Malpighian tubules [138], and nine of the 12 genes encoding molecular V-ATPase subunits’ complex are rhythmically expressed and in a similar phase, each peaking in expression at dusk [18]. One of these, subunit I, has additionally been found to be rhythmic at the protein level [21]. Upregulation of the V-ATPase complex at dusk could thus occur in anticipation of significant osmotic changes induced by a blood or sugar meal, drive a time-of-day-specific ability to cope with osmotic stress and suggest that blood meals taken at inappropriate times-of-day will have negative consequences for the mosquito. This could alter vectorial capacity (Box 1**)** in varying ways. Mosquitoes could be forced to take a smaller blood meal, but may compensate by taking multiple meals (increasing *a^2^*) and, so, exposing themselves to more host defense (decreasing *m* and *a*). They may have difficulty flying away from danger if they cannot excrete water quickly, which increases risk of post-blood feeding mortality (decreases *m* and *P*). Finally, there may be longer-term physiological costs of osmotic stress that reduce survival (*P*). Further experimental work is required to determine if time-of-day indeed contributes to the regulation of osmoregulation.

### 2.7. Rhythms in Immune Factors

Temporal genome profiling of *An. gambiae* [18] revealed rhythmic expression of immune genes that are implicated in modulating malaria infection. This includes genes in the Imd (immune deficiency) and melanization pathways [134,139]. Subsequently, in *An. stephensi*, a time-of-day effect in the expression of immune genes *defensin 1* (*DEF1*), *cecropin 1* (*CEC1*) and *nitric oxide synthase* (*NOS*), has been reported [96]. Phenotypic consequences of rhythms in immune responses have been proposed to explain why the time-of-infection by *E. coli* influences bacteria growth and mosquito mortality [96]. For mosquitoes, rhythms in the actual immune effectors used to defend against infection and/or to control the replication of established parasites are yet to be demonstrated. However, *Drosophila* displays a similar time-of-day effect in post-immune challenge gene expression, and this corresponds to higher survival during *Pseudomonas aeruginosa* infection when flies are challenged in the middle of the night [140]. Finally, the mosquito immune system may also shape the composition of midgut microbiota in a rhythmic manner such as has been demonstrated in other organisms [141]. The composition of the midgut microbiota affects the susceptibility of a mosquito to malaria infection [142] so rhythms in microbiota may result in time-of-day-specific susceptibility to malaria infection.

Assuming there are biologically-relevant consequences of rhythms in immune factors, ROS detoxification (which shapes mosquito immunity, as discussed in the previous sections) and the gut microbiota will all contribute to whether a *Plasmodium*-challenged mosquito develops into an infectious mosquito. Thus, these three components feed into *Vb* of the vectorial capacity equation (Box 1). Additionally, if immune rhythms (or immune challenges at unexpected times-of-day) influence daily survivability, *m* and *P* will be affected.

### 2.8. Further Complexity

We have highlighted reports of suggested or demonstrated mosquito behavioral and blood-meal processing-related phenomena under time-of-day control. These include host-seeking, reactive oxygen species (ROS) detoxification, metabolic detoxification, immunity and osmoregulation. All of these (often interconnected) factors may result in time-of-day-specific consequences to the mosquito from the osmotic and oxidative stress of blood meal digestion, efficient processing of nutrients, the nutrient content of the blood, detoxification of xenobiotics, changes to the gut microbiome of the mosquito, as well as the success of the parasite in establishing an infection in the mosquito. Pleiotropic links in temporal regulation of these processes may constrain the benefits of shifts in biting time in response to bed net use. For example, in *Drosophila*, restricting food intake to the “expected” time-of-day results in improved cardiac function [143]; conversely, restricting food to an “inappropriate” time reduces fecundity [144]. Thus, mosquitoes may be selected to shift the timing of other biological rhythms in concert with shifting biting activity in response to bed nets. The extent to which rhythms can shift to peak at a different time-of-day may also be constrained by exogenous environmental rhythms, such as desiccation risk and predation risk. Clearly, much work is required to disentangle the competing costs and benefits to the mosquito from the temporal regulation of feeding, before the consequences for malaria transmission can be fully understood. Furthermore, biological rhythms in the vertebrate host and malaria parasite add further dimensions of complexity to understanding how time-of-day may shape malaria transmission, and we explore this below.

## 3. Interactions between Mosquito, Host and Parasite Rhythms

### 3.1. Rhythms in Host Blood Composition

Just as mosquito biology is highly rhythmic, so are the genome and physiological processes of human (and other mammalian) hosts that mosquitoes bite [145]. In the contents of a blood meal, there are 24-h rhythms in amino acid levels (with concentrations peaking in the late afternoon) [146], insulin concentration [147,148], immune effectors [149,150] and the oxidative state of red blood cells (RBC) [14]. Mosquitoes will therefore receive a blood meal of differing composition depending on the time-of-day they feed. Variation in the composition of blood contents may affect the “quality” of the blood meal, in terms of its nutrient value and ease of processing. For example, mosquitoes utilize the protein from a blood meal for oogenesis [151], and thus, fecundity may be affected by time-of-day-specific changes in the amino acid composition of blood. Rhythms in the oxidative state of RBC could influence the consequences of ROS damage for the mosquito, parasite and gut microbiota, as proposed above. Finally, human insulin promotes successful parasite development in the mosquito [152], despite increasing oxidative stress for the mosquito [153], by inhibiting the NF-κB-dependent immune response [154]. In humans, insulin concentration follows a 24-h rhythm under controlled conditions (but timing of the rhythm under natural conditions is very much shaped by feeding times, with insulin levels rising after meals [147,148]). It is therefore conceivable that the high level of insulin taken up by mosquitoes feeding on humans shortly after an evening meal could reduce mosquito immune defenses to *Plasmodium* infection [increasing *Vb* in the vectorial capacity equation (Box 1)]; but increasing ROS damage to the mosquito (decreasing *P*). How this affects malaria transmission will depend on the relative contributions of ROS and NF-κB-dependent immunity to both parasite killing and mosquito longevity.

### 3.2. Rhythms in Parasite Infectivity to Mosquitoes

Malaria parasites live in two complex environments, the vertebrate host and the mosquito vector, and must successfully infect and transmit between both. Once injected into a vertebrate host from an infectious mosquito, sporozoites invade liver cells and undergo replication. After several days, the parasite begins its classical blood-stage malaria infection, in which RBC are invaded. Parasites replicate asexually inside RBC in cycles lasting 24, 48 or 72 h (depending on the parasite species), and during every cycle, a small proportion differentiates into sexual stages (gametocytes) that are required for transmission to mosquitoes. Synchronized rhythms in development during asexual cycles have been demonstrated in many *Plasmodium* species infecting humans [155], primates [156], rodents [157,158,159,160,161] and birds [162]. These rhythms are distinctive enough to have been used as a diagnostic feature of the disease, with tertian (recurring every two days; caused by *Plasmodium falciparum*, *ovale* and *vivax*) and quartan malaria (recurring every three days; caused by *Plasmodium malariae*) resulting from the fever/chills associated with the simultaneous bursting of mature parasites from RBC [161,163,164]. Timing of developmental transitions during the asexual cycle seems to be important for parasite fitness, because perturbation relative to the hosts’ circadian rhythm alters parasite life history [165,166,167,168,169] by reducing replication and transmission potential [170,171] and eventual readjustment to match the host rhythm [155,168,172]. However, neither the mechanism (such as a molecular clock) for parasite timekeeping nor entrainment have been identified. It is proposed that rhythmic host metabolism, melatonin levels or nutrient levels [173,174,175] could provide timing cues to the parasite. Additionally, even an exogenous factor, such as light, which parasites have been suggested as responding to [176], could serve as a time cue. Furthermore, the extent to which parasites and hosts are in control of parasite rhythms is poorly understood, though hosts are unlikely to be entirely responsible.

Why parasites may have evolved to synchronize their asexual replication cycles with host circadian rhythm is unknown. Hawking and others have proposed that gametocyte infectivity to mosquitoes is coordinated to the biting time of the vector, reporting rhythms in the production and infectivity of gametocytes in laboratory model systems [6,7,8,9,10,11,12]. However, reports from field studies of natural human malaria infections generally conflict with this idea. Neither rhythms in the density of gametocytes in the blood [177], nor time-of-day variation in the ability of gametocytes to infect *Anopheline* vectors have been observed [178,179]. Another unsupported assumption of Hawking’s hypothesis is that coordinated development of asexual stages is required for coordinated maturation of gametocytes [164]. In contrast, rhythms in asexual cycles can directly impact infectivity. Fever resulting from the simultaneous bursting of mature parasites (schizogony) results in the release of high concentrations of pro-inflammatory cytokines (including TNF-α and IFN-γ), which can damage gametocytes and prevent transmission for several hours [180,181]. Furthermore, because the human immune system responds to infection in a time-of-day-specific manner [149,150], rhythms in immune responses could alter the infectiousness of gametocytes in a time-of-day-specific manner. In addition to their effects on gametocytes, fever rhythms in the host may reinforce the synchronization of asexual cycles by killing slow developing parasites [182,183,184,185]. Separating the extent to which rhythms in infectivity are a cause and consequence of endogenous rhythms in parasite asexual cycles and interactions with host immune defenses is non-trivial. Unfortunately, this information is required to parameterize the part of the vectorial capacity equation (Box 1) characterizing the success of parasites at establishing infection in the mosquito (*V*) and predicting whether parasites are disadvantaged (or facilitated) by, for example, a shift in mosquito rhythms to day-time biting.

### 3.3. Rhythms in Infectivity to Hosts

Once infection is established in the vector, parasites undergo several weeks of replication to produce many thousands of sporozoites. Sporozoites migrate *en masse* to the salivary glands, where they remain until injected into a host when the mosquito takes the next blood meal. The time period required for the parasite to mature, known as the extrinsic incubation period (EIP; the *n* term in the vectorial capacity equation; see Box 1), is temperature dependent. For example, *Plasmodium falciparum* is predicted to take ~10 days to mature at ~30 °C or ~25 days at 20 °C [186]. Very little is known about the within-vector processes that influence the production, migration and infectiousness of sporozoites, but there are plenty of opportunities for rhythms to be involved. For example, the migration of sporozoites from the hemocoel to the salivary glands (where they accumulate) could be rhythmic and timed to occur at particular times-of-day when mosquito immune cells (e.g., hemocytes) are least active/numerous. Due to the concern that climate change may facilitate malaria transmission, a topic receiving increasing interest is how the exogenous daily environmental temperature rhythm affects EIP. EIP was classically thought to be determined simply by the temperature-dependent nature of biochemical reactions and assessed/modeled using daily average temperatures [113,186]. However, it is now understood that ignoring daily temperature rhythms can result in a poor estimate of EIP, because daily means can mask times-of-day when temperature deviates from the range when parasite development can proceed [102,103]. Note a temperature-driven growth development rate of the parasite does not exclude the possibility of a circadian clock/rhythms (whose time keeping is compensated for temperature variation). For example, the division rate of the cyanobacterium, *Synechococcus*, is both temperature and time-of-day dependent [187,188].

In Section 2, we highlighted how the overlap in mosquito and host behavioral rhythms affects the success of mosquito biting (this is reflected in the vectorial capacity equation as *ma*). However, rhythms in other physiological processes of the parasite and mammalian host could contribute to the parasite successfully getting “out” of the mosquito and establishing an infection in the human host. Important components of the mammalian immune system are rhythmic [149,150], but whether these rhythms persist during malaria infections is unknown. However, any rhythms in innate defenses that prevent invasion by parasites are likely to make mammalian hosts susceptible to infection in a time-of-day-specific manner. Conversely, the sporozoites may be more infectious at certain times-of-the day, for example, by upregulating immune system-evading processes at night when it is more likely the mosquito will take a blood meal. Further work is required to determine if either of these phenomena occur. When we consider the vectorial capacity equation (Box 1), host factors that affect the success of parasites making the transition from vector to host are important determinants of *Vb.* Given that the rhythms discussed here could interact in complex ways with rhythmic processes discussed in Section 3.2 to determine *Vb* and *n*, a better understanding of within-vector ecology is urgently required.

## 4. The Impact of Rhythms for Interventions

Humans are responsible for considerable changes to our environment, some of which affect circadian biology. Building protective dwellings, timing of insecticidal fogging and reductions in mosquito predators (e.g., bat habitat destruction) are all means by which humans are changing mosquito temporal selective pressures. Bed nets have been extremely successful at reducing malaria transmission [189], because they harness the temporal overlap between mosquito (foraging) and human (sleeping) behaviors. Unfortunately, the evolution of day-biting behavior is occurring, and so, much of our discussion has centered on the implications of this, through interactions with rhythms in other vector processes, hosts and parasites, for malaria transmission. The importance of rhythms for vectorial capacity also offers the opportunity for novel interventions, such as specific timing of insecticidal fogging or the development of intervention techniques that target mosquitoes active only at specific times-of-day (e.g., a baited trap that is only activated at times-of-day when humans are not protected under bed nets would counteract the selective pressure of bed nets). Similarly overlooked, the increasing use of artificial light may be affecting transmission in two ways. First, enabled by artificial lights, societal shifts to more nocturnal activity may increase the overlap in active periods between humans and mosquitoes, offering more transmission opportunities to parasites. Second, light itself can modulate the behavior and physiology of the mosquito, including biting behavior, foraging behavior and gene expression (see Table 1). Thus, as a consequence of modern day environmental light pollution, or as a yet undeveloped intervention strategy, light may alter malaria transmission by modulating biting rate (*ma*), the long-term survivability of the mosquito (*P*) or possibly even the success of parasite establishment (*Vb*).

## 5. Conclusions

We have summarized various ways that mosquito circadian biology and time-of-day may interact with hosts and parasites to shape malaria transmission. We have limited our discussion to daily 24-h rhythms, but longer rhythms, such a lunar and seasonal rhythms, are also acting on malaria transmission and should not be ignored [45,190]. Behavioral rhythms in foraging and biting times are the most well investigated, but we highlight that rhythms in olfactory sensitivity and other behaviors are likely to be important. We also suggest that the potential for rhythms in mosquito immunity, reactive oxygen species detoxification, osmotic regulation and metabolic detoxification require attention. The temporal selective pressures exerted by bed nets [36,42] are intuitively expected to change mosquito biting time, but constraints on rhythms in other aspects of mosquito biology, and conflict between rhythms in parasites and hosts, make the net consequences of day biting for malaria transmission hard to predict. It is clear that vectorial capacity is likely to exhibit daily cycles because a high proportion of the parameters may be time-of-day-dependent. However, the model for vectorial capacity assumes independence among terms that are likely tightly coupled (for instance, the daily survival rate affects the number of mosquitoes per host). Therefore, further analysis and perhaps a more refined modeling effort are required to quantify rhythms in vectorial capacity.

## Figures and Tables

**Table 1:** Time-of-day affects many aspects of mosquito biology.

Mosquito Rhythms in:	Anophelines	Other Mosquito Species
Insecticide response	[24]	[25] *, [26,27]
Vision	[28,29]	[30,31]
Olfaction	[21]	
Biting behavior (including bed net use and biting time)	[21] *, [32,33,34,35,36,37,38,39,40,41,42,43]	[22,39,40,44,45]
Molecular clock genes	[18,46] *	[19,20] *, [47,48,49,50,51,52,53]
Genome-wide transcriptomics	[18,23,46] *	[20] *, [54]
Oviposition	[55,56,57,58]	[59,60]
Locomotor flight activity	[61,62,63,64,65,66,67] *, [68,69,70,71]	[72,73,74,75,76] *, [31,70,71,77,78]
Mating	[67] *, [79,80,81]	[73,82] *, [77,83]
Larval/pupal rhythms	[65,84] *	[85] *, [26,27,86,87]
Sugar feeding	[78,88]	[89] *, [78]
Metabolism	[90]	[85] *, [86,87,91,92]
Cuticle development	[93]	[94]
Predation risk	[95]	
Immunity	[96]	
**Related Work with Time-of-Day Aspects:**		
Diapause induction		[97,98]
Behavioral changes during infection	[99]	[99,100,101]
Environmental temperature rhythms	[102,103]	[104]
The role and effect of light and the light:dark cycle	[46,63,66,68,105,106]	[74,76,107,108]

* The reference(s) provides evidence of an endogenous (circadian) mosquito rhythm.
